# Comparison of genetic and epigenetic profiles of periodontitis according to the presence of type 2 diabetes

**DOI:** 10.1002/mco2.620

**Published:** 2024-06-19

**Authors:** Junho Kang, Hansong Lee, Ji‐Young Joo, Jae‐Min Song, Hyun‐Joo Kim, Yun Hak Kim, Hae Ryoun Park

**Affiliations:** ^1^ Department of Research Keimyung University Dongsan Medical Center Dalseo‐gu Daegu Republic of Korea; ^2^ Department of BioMedical Informatics Medical Research Institute, Pusan National University Yangsan‐si Gyeongsangnam‐do Republic of Korea; ^3^ Department of Periodontology School of Dentistry Pusan National University Yangsan‐si Gyeongsangnam‐do Republic of Korea; ^4^ Department of Oral and Maxillofacial Surgery School of Dentistry Pusan National University Yangsan‐si Gyeongsangnam‐do Republic of Korea; ^5^ Department of Periodontology Dental and Life Science Institute School of Dentistry Pusan National University Yangsan‐si Gyeongsangnam‐do Republic of Korea; ^6^ Department of Periodontology and Dental Research Institute Pusan National University Dental Hospital Yangsan‐si Gyeongsangnam‐do Republic of Korea; ^7^ Periodontal Disease Signaling Network Research Center School of Dentistry Pusan National University Yangsan‐si Gyeongsangnam‐do Republic of Korea; ^8^ Department of Biomedical Informatics School of Medicine Pusan National University Yangsan‐si Gyeongsangnam‐do Republic of Korea; ^9^ Department of Anatomy School of Medicine Pusan National University Yangsan‐si Gyeongsangnam‐do Republic of Korea; ^10^ Department of Oral Pathology School of Dentistry Pusan National University Yangsan‐si Gyeongsangnam‐do Republic of Korea

**Keywords:** diabetes mellitus, EPIC array, Fc‐gamma receptor, periodontitis, whole genome sequencing

## Abstract

Type 2 diabetes mellitus (T2DM) and periodontitis (PD) have intricated connections as chronic inflammatory diseases. While the immune response is a key factor that accounts for their association, the underlying mechanisms remain unclear. To gain a deeper understanding of the connection, we conducted research using a multiomics approach. We generated whole genome and methylation profiling array data from the periodontium of PD patients with DM (PDDM) and without DM to confirm genetic and epigenetic changes. Independent bulk and single‐cell RNA sequencing data were employed to verify the expression levels of hypo‐methylated genes. We observed a gradual rise in C>T base substitutions and hypomethylation in PD and PDDM patients compared with healthy participants. Furthermore, specific genetic and epigenetic alterations were prominently associated with the Fc‐gamma receptor‐mediated phagocytosis pathway. The upregulation of these genes was confirmed in both the periodontal tissues of PD patients and the pancreatic tissues of T2DM patients. Through single‐cell RNA analysis of peripheral blood mononuclear cells, substantial upregulation of Fc‐gamma receptors and related genes was particularly identified in monocytes. Our findings suggest that targeting the Fc‐gamma signaling pathway in monocytes holds promise as a potential treatment strategy for managing systemic complications associated with diabetes.

## Graphical abstract

Periodontitis accompanied by type 2 diabetes exhibits significant hypomethylation of Fc gamma receptor‐related genes compared with periodontitis.

Periodontitis accompanied by type 2 diabetes shows a notable increase in C>T base substitutions within Fc gamma receptor‐related genes compared with periodontitis.

Hypomethylation and C>T base substitutions display a linear correlation, with a stronger correlation observed in periodontitis accompanied by type 2 diabetes.

Fc gamma receptor‐related genes are significantly upregulated in monocytes of patients with periodontitis accompanied by type 2 diabetes.

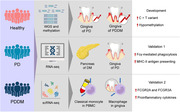

## 1 INTRODUCTION

Type 2 diabetes mellitus (T2DM) is a metabolic disorder distinguished by impairments in either insulin secretion, insulin action, or both.^1^ T2DM represents the predominant form, encompassing 90% of all cases of diabetes mellitus (DM) and it results from inefficient utilization use of insulin.^2^ The global incidence of DM has significantly increased in the recent decades. The number of cases have risen from 108 million in 1980 to 422 million in 2014, and the projections indicate that the number of cases will more than double in the next 20 years.^3^ The World Health Organization has predicted that by 2030, diabetes will become the seventh leading cause of death. As one of T2DM complications, periodontitis (PD) is a widely recognized disease. PD affects the tissues surrounding the teeth and is characterized by gingivitis, periodontal attachment loss, alveolar bone resorption, and eventual tooth loss.^4^ PD, ranked the sixth most common chronic disease, affects approximately 750 million people worldwide and significantly impacts chewing ability, nutritional status, and quality of life.^5^

Numerous studies have established a bidirectional relationship between T2DM and PD.^6^, ^7^ PD can be worsened by T2DM, and conversely, PD can exacerbate glycemic control and elevate the risk of diabetic complications.^8^ T2DM contributes to the initiation and progression of PD through mechanisms such as hyperinflammatory response, impaired bone healing processes, and increased production of advanced glycation end products.^9^ In addition, PD can persistently elevate systemic levels of certain substances like interleukin (IL)‐6, tumor necrosis factor (TNF)‐α, and C‐reactive protein (CRP), leading to systemic inflammation.^10^, ^11^, ^12^ Consecutive systemic inflammation can exert detrimental effect on hematopoietic stem progenitor cells and eventually trigger DNA damage, hyperactivity of myeloid cells, and organ dysfunctions.^13^, ^14^, ^15^, ^16^, ^17^ As another bridge between T2DM and PD, recent insights into epigenetic regulation suggest a significant role in the diabetic pathways. Epigenetic changes, including DNA methylation, histone modifications, and noncoding RNA interactions, have been linked to both the onset and progression of T2DM and PD.^18^ These modifications can result in unresolved inflammation and have lasting impact on cellular responses independent of the environmental stimulus.^19^ Thus, the epigenetic changes exacerbates the inflammatory response and leads to pathological gene expression, which can influence insulin signaling pathways, contributing to the complex interplay between T2DM and PD.^20^, ^21^

Although there are possible mechanisms playing crucial role in connecting PD and T2DM, there is few research conducted through a multiomics approach that can provide a more holistic perspective of the complex interactions among genes, transcriptomes, and proteins. Therefore, to achieve a more comprehensive understanding of linkage between T2DM and DM, in the present study, we generated and applied multiomics data to look into the genetic and epigenetic link between PD and T2DM and develop the novel therapeutic targets.

## 2 RESULTS

### 2.1 Clinical characteristics and cohort descriptions

Our study comprised a total of 36 patients, divided into three groups: 10 healthy individuals, who served as the reference group in all analyses, 15 patients with PD, and 11 patients with PD and type 2 diabetes mellitus (PDDM). While there were no notable differences observed in acute inflammatory indicators such as CRP and erythrocyte sedimentation rate (ESR) among PD and PDDM, both PD and PDDM patients exhibited higher age on average than that of healthy individuals (Table 1). The hemoglobin A1c (HbA1c) levels were elevated exclusively in PDDM patients, while the body mass index (BMI) ranges remained within the normal scale for both PD and PDDM. These findings suggest that individuals in the PDDM group manifested DM independent of obesity.

**TABLE 1:** Characteristics of included patients depending on group.

Group	Healthy	PD	PDDM
Patients, *n*	10	15	11
Age (mean, SD), years	37.30 (16.91)	54.47 (11.11)^a^	65.82 (13.43)^a^
Men:women	10:0	6:9	7:4
BMI (mean)	NA	24.95	23.29
BMI, *n*			
<20.5 (low weight)		0	1
20.5–25.5 (normal weight)		7	7
25.5–30 (overweight)		4	1
≥30 (obese)		1	0
NA	10	3	2
HbA1c (mean)	5.18	5.51	7.30^a^
HbA1c, *n*			
<5.7%	9	9	1
5.7–6.5%	1	5	3
≥6.5%		0	7
NA	0	1	0
ESR, mean (mm/h)	4.20	8.35	4.80
ESR, *n* (%)			
<15 mm/h	10	11	10
15−20 mm/h	0	1	0
>20 mm/h	0	2	0
NA (*n*)	0	1	1
CRP, mean (mg/L)	0.99	0.79	1.31
CRP, *n* (%)			
<0.5 mg/L	7	6	5
0.5–3.0 mg/L	2	9	5
≥3.0 mg/L	1	0	1
NA (*n*)	0	0	0

^a^ Wilcoxon rank sum test result indicating statistical significance compared with healthy group (*p* value < 0.05). The comparisons were conducted on age, BMI, HbA1c, ESR, and CRP measurements.

### 2.2 Differences in somatic mutation patterns between patients with PD and PDDM

**FIGURE 1:**
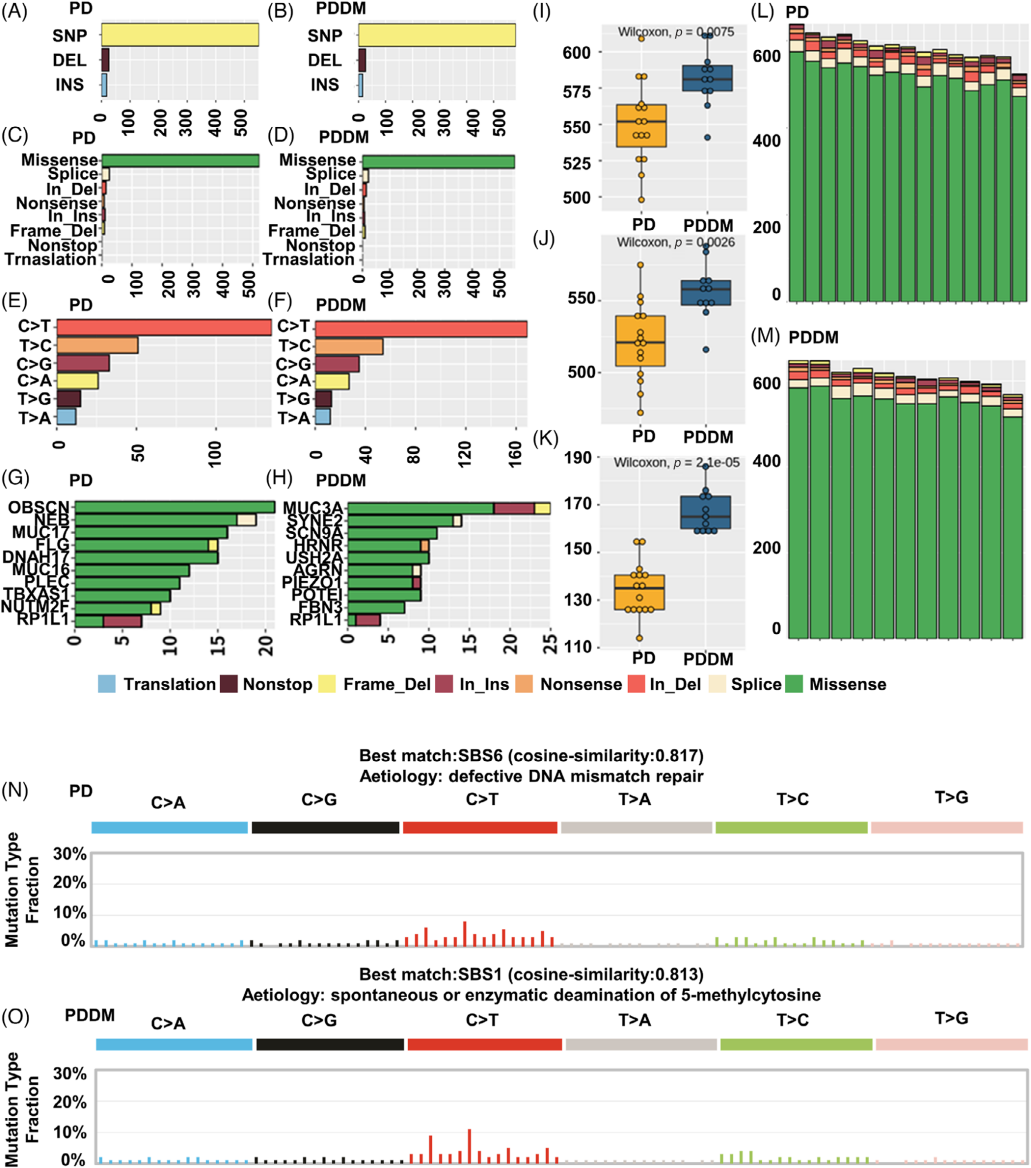
Differences in mutation patterns between patients with periodontitis and those with periodontitis and diabetes. (A) Indicates the variant burden of patients included in the PD group. The *y*‐axis represents the number of all the variants. The color of each bar follows the legend shown on the right side, and each color represents a variant type. (B) Variant burden of patients included in the PDDM group. The *y*‐axis represents the number of all the variants. (C) Variant types of the top 10 most frequently mutated genes in the PD group. The *x*‐axis represents the mutations contained in the gene, and the *y*‐axis represents the gene symbol. (D) Variant types of the top 10 most mutated genes in the PDDM group. (E) Burden according to variant type in PD group. The *x*‐axis represents the number of occurrences of each variant type, and the *y*‐axis represents the variant type. (F) Burden according to variant type in PDDM group. (G) SNP *t*‐test results from PD and PDDM groups. The *x*‐axis represents each group, and the *y*‐axis represents the number of SNPs. (H) Burden according to variant class in the PD group. The *x*‐axis represents the number of occurrences of each class, and the *y*‐axis represents the class. (I) Burden according to variant class in the PDDM group. (J) Missense mutation *t*‐test results in PD and PDDM groups. The *x*‐axis represents each group, and the *y*‐axis represents the number of missense mutations. (K) Burden according to base substitution in the PD group. The *x*‐axis represents the number of each base substitution, and the *y*‐axis represents base substitution. (L) Burden according to base substitution in the PDDM group. (M) *t*‐Test result of C>T tooth replacement in PD and PDDM groups. The *x*‐axis represents each group, and the *y*‐axis represents the number of C > T base substitutions. (N) Mutational signature with the highest similarity in the PD group. Each color represents a matching base substitution, the *x*‐axis represents 96 base substitutions, and the *y*‐axis represents the percentage of each base substitution. (O) Mutational signature with the highest similarity in the PDDM group.

**FIGURE 2:**
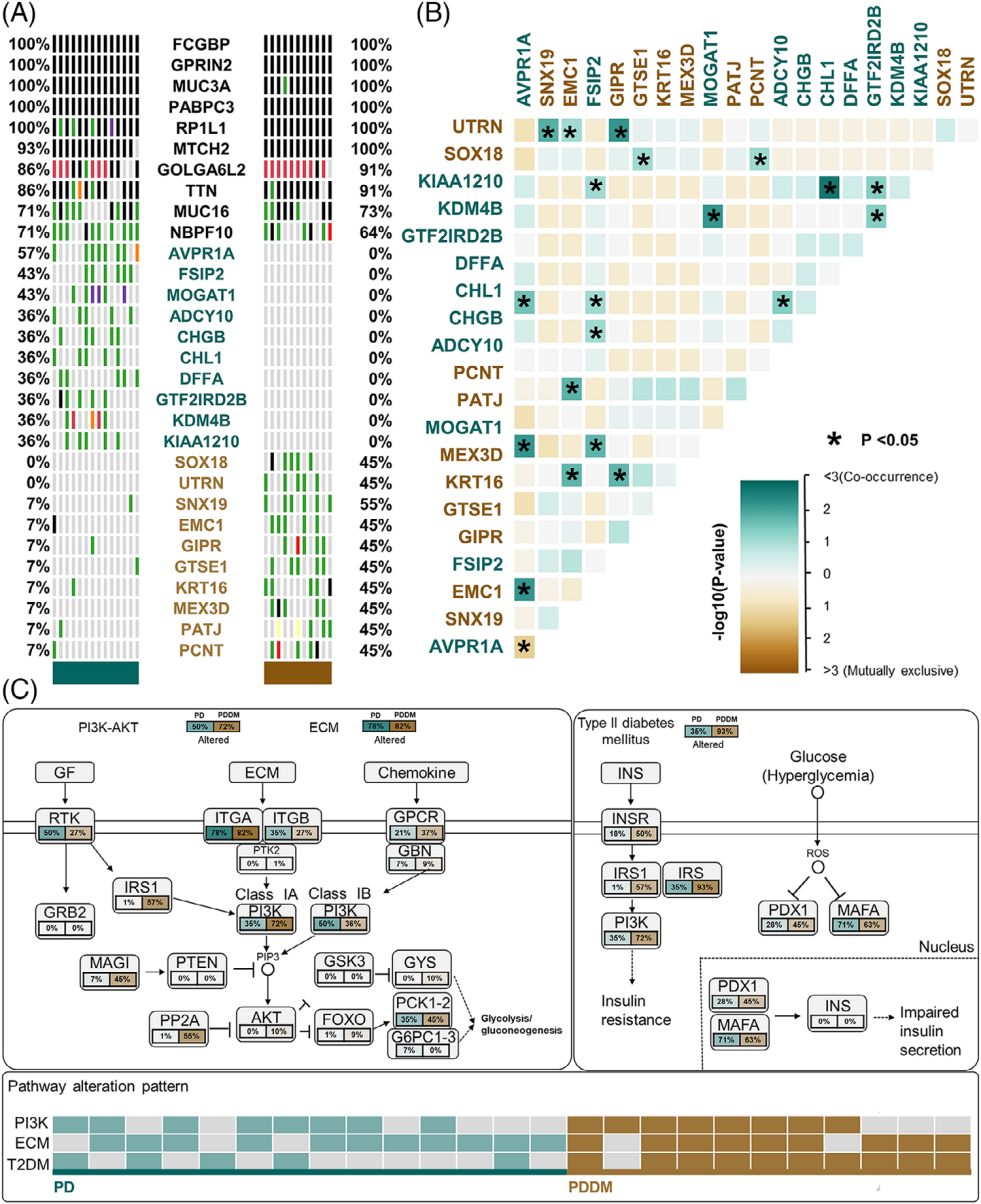
Characteristics of somatic mutations in patients with PD and PDDM. (A) Oncoplot showing common mutated genes and specific mutated genes in patients with PD and PDDM. The left side of the oncoplot represents the PD group, while the right side represents the PDDM group. The percentages displayed on the left side indicate the proportion of patients carrying mutations in that gene in the PD group, and the percentages on the right side indicate the proportion of mutations in the PDDM group. The gene symbols are represented in the middle of the panel. (B) Somatic interaction between specific genes. The color of each cell indicates the type of interaction. Cyan represents co‐occurrence, while brown represents mutual exclusivity. (C) Mutation patterns in the top three signaling pathways of genes with high number of mutations. The percentage indicated below each gene represents the proportion of patients with a mutation in that gene among all patients, with red indicating a higher proportion. Solid lines represent the cell membrane, dotted lines represent the nuclear membrane. Arrows indicate activation, bars represent inhibition, and dotted arrows represent indirect effects of the stated changes. In the pathway alteration pattern, each cell represents one patient, with red color indicating the presence of at least one genetic alteration in the pathway.

Patients with PD and PDDM exhibit notable differences that contribute to the exacerbation of PD and increase inflammatory responses.^22^, ^23^ To compare the mutation patterns, we performed a somatic mutation analysis between patients with PD and PDDM. The mean number of mutations was 564 (534–617) in patients with PD, while it was 589 (545–647) in patients with PDDM, indicating a higher average number of mutations in patients with PDDM (Figure 1A,B). In patients with PD, the highly mutated genes included *OBSCN*, *NEB*, *MUC17*, and *FLG*, whereas in patients with PDDM, these were *MUC3A*, *SYNE2*, *SCN9A*, and *HRNR* (Figure 1C,D). These genes were characterized by their considerable length, potentially contributing to the occurrence of common polymorphisms within them. A comprehensive list of mutations found in patients with PD and PDDM is included in Tables S1 and S2. We analyzed the differences between various mutation types, including single nucleotide polymorphisms (SNPs), insertion and deletion (INDEL), and base substitutions, between the two groups. The results revealed no difference in INDEL between the groups (Figure 1E,F); however, patients with PDDM exhibited a significantly higher number of SNPs compared with those with PD (*p *= 0.0075) (Figure 1G). Additionally, missense mutations were significantly common in patients with PDDM, while no differences were observed in terms of other mutation types (*p *= 0.0026) (Figure 1H–J). The most common based substitution event observed was C>T. Notably, patients with PDDM exhibited a significantly higher number of C>T substitutions compared with that of patients with PD (*p *= 2.1^e−07^) (Figure 1K–M). In summary, patients with PDDM exhibited significantly higher number of mutations compared with those with PD, with a notable increase in the number of C>T substitutions at single nucleotide level. Additionally, we performed mutational signature analysis based on single nucleotide substitution patterns. Patients with PD exhibited the highest similarity to single base substitution signature (SBS) 6 (cosine similarity: 0.817) (Figure 1N), while those with PDDM exhibited the highest similarity to SBS1 (cosine similarity: 0.813) (Figure 1O). Interestingly, patients with PDDM exhibited more C>T single nucleotide substitutions than patients without PDDM, which were most similar to the SBS1 signature. SBS1 is linked to the natural mutation process triggered when 5‐methylcytosine undergoes spontaneous or enzymatic deamination, resulting in its conversion to thymine.^24^ These results suggest that the increased number of C>T base substitutions in patients with PDDM could be due to spontaneous mutations of 5‐methylcytosine. We examined mutation patterns not only at the mutation type level but also at the gene level. Common mutated genes between the two groups included *FCGBP*, *GPRIN2*, *MUC3A*, and *PABPC3*. PD‐specific gene mutations included *AVPR1A* (PD:57% vs. PDDM:0%), *FSIP2* (PD:43% vs. PDDM:0%), *MOGAT1* (PD:43% vs. PDDM:0%), and *ADCY10* (PD:36% vs. PDDM:0%), whereas PDDM‐specific gene mutations included *SNX19* (PDDM:55% vs. PD:7%), *SOX18* (PDDM:45% vs. PD:0%), and *UTRN* (PDDM:45% vs. PD:0%) (Figure 2A). The gene with the highest co‐occurrence in PD‐specific gene mutations was *FSIP2*, which co‐occurred with five out of 10 genes. In PDDM‐specific gene mutations, *GIPR* significantly co‐occurred with two out of ten genes, and *SNX19* and *AVPR1A* mutations were mutually exclusive (Figure 2B). Though the role of FSIP2 in PD remains unclear, *FSIP2* exhibits immunomodulatory functions in skin cutaneous melanoma.^25^ Additionally, SNPs in the *GIPR* gene have been associated with alterations in hormone and adipokine secretion in obese type 2 diabetic patients.^26^, ^27^ We evaluated pathways with a high mutation burden in both the groups, focusing on frequently mutated genes involved in signaling. The top three pathways included PI3K–AKT, extracellular matrix, and T2DM (Table S3). Patients with PDDM exhibited a higher mutation burden than that of the patients with PD (Figure 2C). Previous studies on diabetes have suggested that functional impairments in certain pathways, particularly the PI3K–AKT pathway, play a role in hyperglycemia and insulin resistance.^28^

### 2.3 Genome‐wide methylation patterns analysis

**FIGURE 3:**
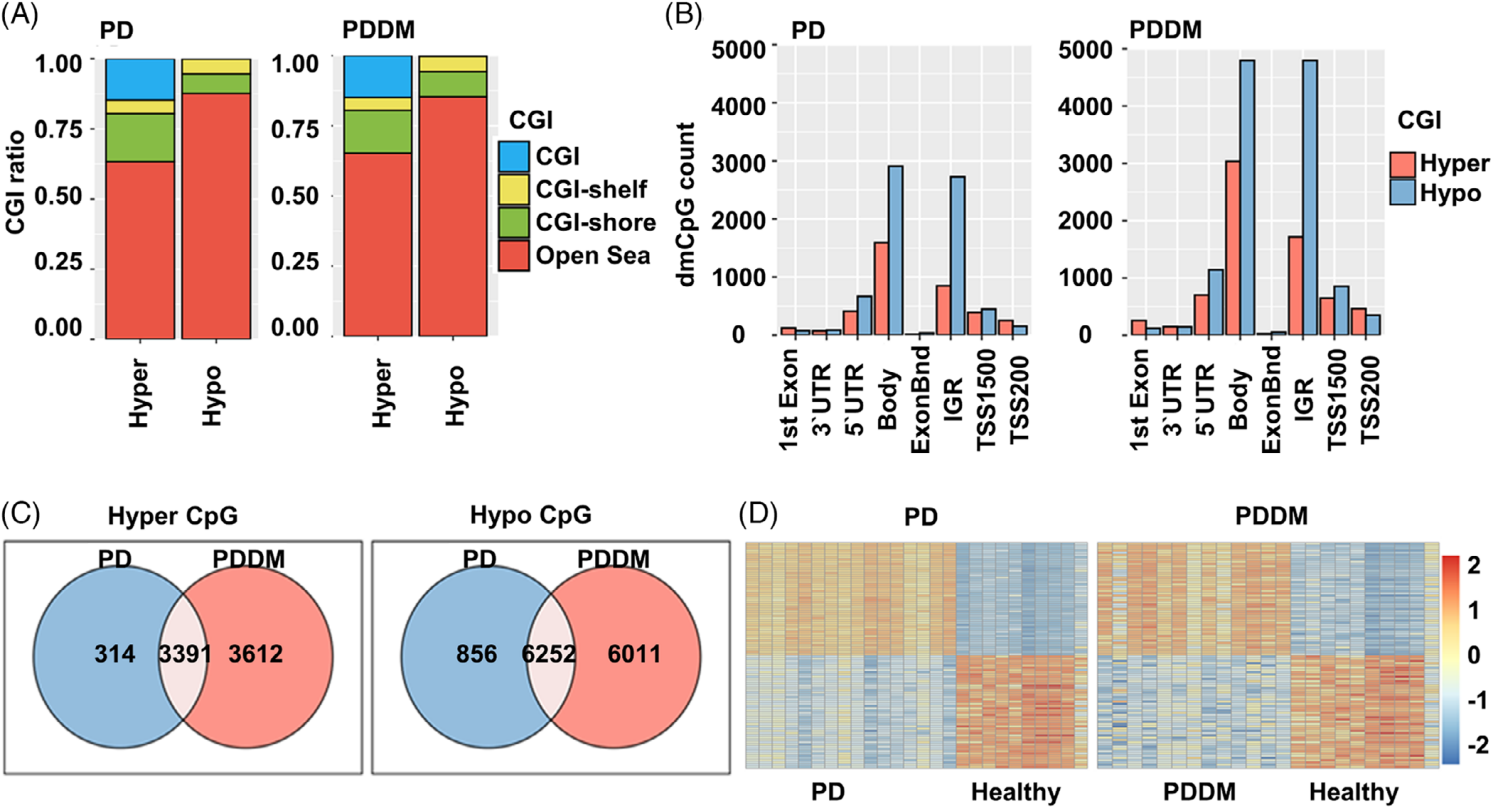
Differences of methylation patterns between patients with PD and PDDM. (A): Barplot depicting the distribution of CpG island locations. The *x*‐axis represents hypermethylation and hypomethylation, while the *y*‐axis represents the proportion occupied by each region. Each color corresponds to the legend displayed on the right side. (B) Barplot illustrating the detailed locations of the CpG island. The *x*‐axis represents each position, and the *y*‐axis represents the number of dmCpG. The color of each bar is red for hypermethylation and blue for hypomethylation. (C) Venn diagram displaying the overlapping dmCpG sites between patients with PD and PDDM. Blue color represents patients with PD, and the red color represents patients with PDDM. (D) Heatmap showing the dmCpG in PD and PDDM groups. The *x*‐axis represents each group, while the *y*‐axis represents dmCpG sites. The color of each cell indicates the intensity of differential methylation, with red indicating hypermethylation and blue indicating hypomethylation.

**FIGURE 4:**
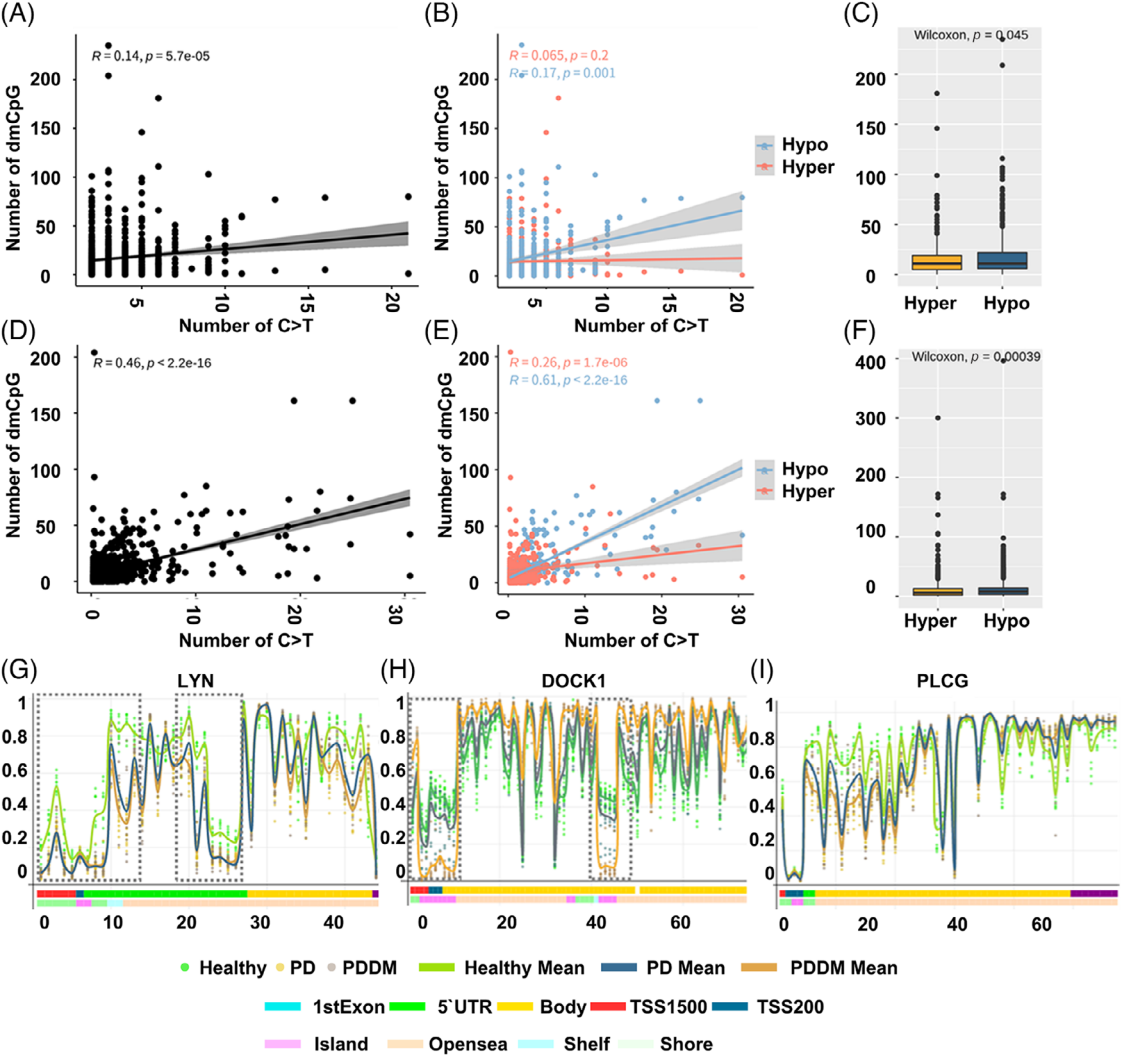
Correlation analysis between C>T base substitutions and dmCpG sites. (A) Correlation analysis between C>T base substitutions and dmCpGs in the PD group. The *x*‐axis represents the number of C>T base substitutions per gene, and the *y*‐axis represents the number of dmCpG. (B) Correlation analysis based on hyper‐dmCpG and hypo‐dmCpG in the PD group. Red indicates hyper‐dmCpG, and blue indicates hypo‐dmCpG. (C) Differences in hyper‐dmCpG and hypo‐dmCpG in genes containing C>T base substitutions in the PD group. The *x*‐axis represents the methylation state, and *y*‐axis represents the number of occurrences for each state. (D) Correlation analysis between C>T base substitutions and dmCpG in the PDDM group. The *x*‐axis represents the number of C>T base substitutions per gene, and the *y*‐axis represents the number of dmCpG. (E) Correlation analysis based on hyper‐dmCpG and hypo‐dmCpG in the PDDM group. Red indicates hypermethylation, and blue indicates hypomethylation. (F) Differences in hyper‐dmCpG and hypo‐dmCpG in genes containing C>T base substitutions in the PDDM group. The *x*‐axis represents the methylation state, and the *y*‐axis represents the number of occurrences for each state. (G) Gene plot of the *LYN* gene. Each line and color correspond to the legend displayed on the right side. The *x*‐axis represents the absolute position in the gene, and the *y*‐axis represents the methylation intensity. Dotted lines indicate regions where C>T base substitutions and hypomethylation coincide. (H) Gene plot of the *DOCK1* gene. (I) Gene plot of the *PLCG* gene.

**FIGURE 5:**
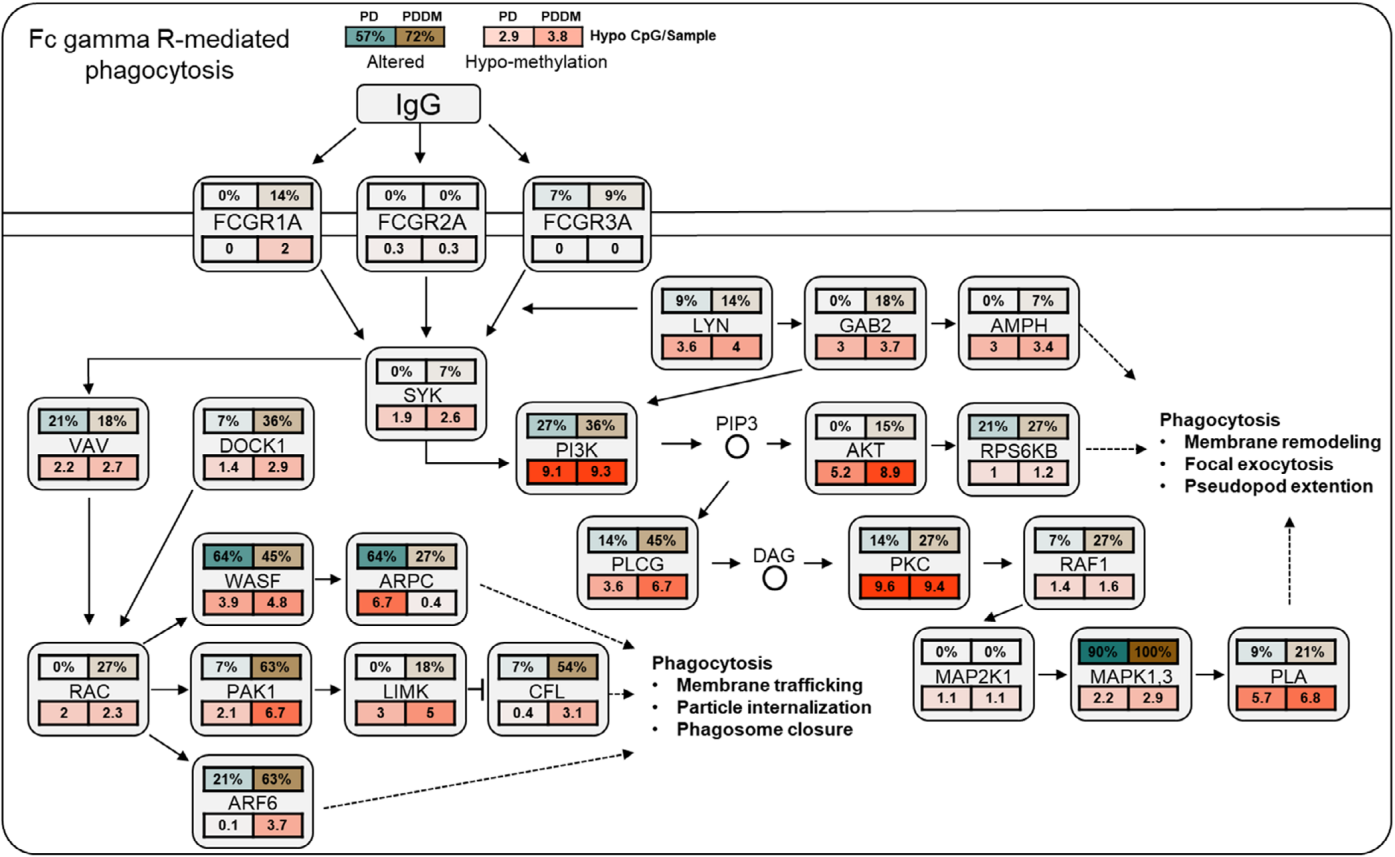
Difference between C>T base substitutions and hypomethylation in the Fc‐gamma receptor‐mediated phagocytosis signaling pathway. The percentages displayed above each gene indicate the proportion of patients with mutations in that gene out of the total number of patients. Turquoise color represents patients with PD, and brown color represents those with PDDM. The percentages shown below each gene represent the number of dmCpG per patient, with increasing intensity indicated by red color. The percentage next to each pathway represents the proportion of patients with at least one mutation in one of the genes involved in that pathway. Solid lines represent the cell membrane, while dotted lines represent the nuclear membrane. Arrows indicate activation, bars indicate suppression, and dotted arrows represent the indirect effects of the stated change.

According to previous studies, there exists a strong correlation between C>T mutations and methylation at CpG dinucleotides.^29^, ^30^ To inspect the relationship between the mutation and methylation patterns, thus, we conducted a genome‐wide methylation analysis for both the groups. The distribution of differentially methylated CpGs (dmCpGs) in both the groups revealed a higher proportion of hyper dmCpG in the order of open sea, shore, island, and shelf. Similarly, hypo dmCpG followed the same order but showed minimal distribution on the island (Figure 3A). Upon further refinement, the gene body and integrin regions exhibited the highest number of dmCpGs (Figure 3B). We identified 10,813 dmCpGs in the PD group (hyper dmCpG: 3705, hypo dmCpG: 7108) and 19,266 (hyper dmCpG: 7003, hypo dmCpG: 12,263) in the PDDM group, approximately double the number (Figure 3C). A heatmap of dmCpG identified in each group is shown in Figure 3D. Notably, the PDDM group exhibited a higher number of C>T mutations and hypo dmCpG than those of the PD group. Spontaneous deamination of methylated cytosine is a well‐known mutation hotspot, particularly associated with hypo methylation.^31^ Based on these findings, we conducted a correlation analysis at the gene level for C>T base substitutions and dmCpGs to ascertain their correlation. As a result, we identified a significant correlation between C>T base substitutions and dmCpG in the PD group (*p *= 5.7^e−05^) (Figure 4A; *R* = 0.14). We observed no significant linearity with hyper dmCpG (Figure 4B; *R* = 0.065, *p *= 0.2); however, a significant linearity was observed with hypo dmCpG (Figure 4B; *R* = 0.17, *p *= 0.001). Moreover, we observed a significantly higher presence of hypo dmCpG than hyper dmCpGs in genes containing C>T base substitutions (*p *= 0.045) (Figure 4C). In the PDDM group, a significantly higher association was observed between C>T base substitutions and dmCpG compared with those of the PD group (*R* = 0.46, *p *= 2.2^e−16^) (Figure 4D). Although both hyper and hypo dmCpGs demonstrated significant linearity, hypo dmCpG (*R* = 0.61, *p *= 2.2^e−16^) (Figure 4E) displayed a stronger linearity than hyper dmCpG in the PDDM group (*R* = 0.26, *p *= 1.7^e−06^) (Figure 4E). Based on these findings, we established a strong correlation between hypo dmCpG and C>T base substitutions, and we further conducted a functional enrichment analysis focusing on genes exhibiting both C>T base substitutions and hypo dmCpG. The functional enrichment analysis confirmed that these genes were the most highly concentrated in the Fc‐gamma R‐mediated phagocytosis signaling pathway. Detailed results of the functional enrichment analysis are presented in Table S4. The Fc‐gamma receptors are membrane receptors of various immune cells. In genes exhibiting C>T base substitutions, a significantly higher proportion of hypo dmCpGs were observed compared with hyper dmCpGs (*p *= 0.00039) (Figure 4F). Interestingly, genes such as *LYN*, *DOCK1*, *PLCG*, *MYO10*, *AMPH*, *INPP5D*, and *PRKCA* exhibited C>T base substitutions and hypo methylation from the transcription start site 1500 (TSS1500) to the 1st exon region (Figures 4G–I and S1). The burden of C>T base substitutions in this pathway was 57% in the PD group and 72% in the PDDM group, with a mean hypo methylation intensity of 2.9 and 3.8 in PD and PDDM groups, respectively (Figure 5). This finding suggests the infiltration of immune cells with genetic alterations in Fc‐gamma receptor‐related genes in the periodontal tissues of PD and PDDM patients.

### 2.4 Validation of the results in independent bulk RNA sequencing datasets

**FIGURE 6:**
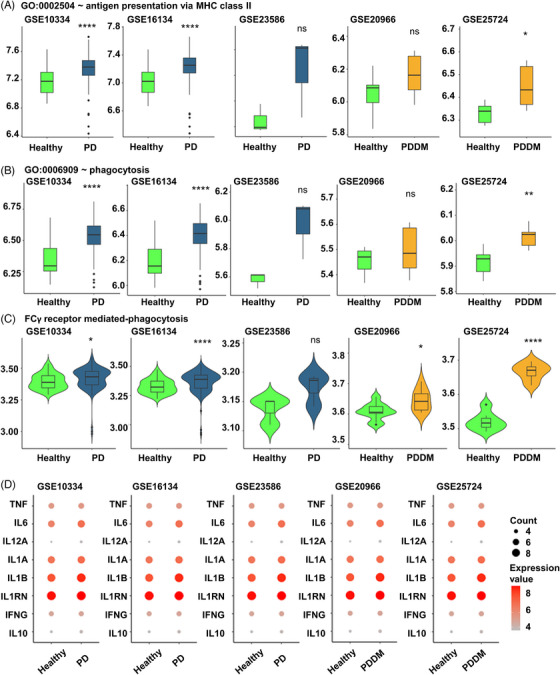
Expression of genes related to Fc‐gamma receptor‐mediated phagocytosis in bulk tissue. (A) Changes in expression values of genes involved in antigen presentation via MHC class 2. The *x*‐axis represents the groups in each cohort, and the *y*‐axis represents the expression value. (B) Changes in expression values of genes involved in phagocytosis. The *x*‐axis represents the groups in each cohort, and the *y*‐axis represents the expression value. (C) Changes in expression values of genes involved in Fc‐gamma receptor‐mediated phagocytosis. The *x*‐axis represents the groups in each cohort, and the *y*‐axis represents the expression value. (D) Dot plot of inflammatory cytokine expression values. The color of each dot represents the relative expression value, with red indicating a relatively high expression value. The size of the dots represents the absolute expression value. The horizontal line represents average expression for each group and star notation demonstrates significance of difference based on Wilcoxon rank sum test. *p* ≤ 0.05 (*), *p* ≤ 0.01 (**), *p* ≤ 0.001 (***), *p* ≤ 0.0001 (****), *p* ≥ 0.05 (ns).

Hypo methylation in the TSS200–TSS1500 region of Fc‐gamma receptor‐mediated phagocytosis signaling pathway could potentially influence the expression of the corresponding genes. We analyzed the gingival tissues of patients with PD (GSE10334, GSE16134, and GSE23586) and pancreatic tissues of patients with DM (GSE20966 and GSE25724) to verify our findings. Antigen presentation via major histocompatibility complex (MHC) class 2 and phagocytosis related genes of all datasets were found to increase in diseased lesions compared with healthy controls (Figure 6A–C). Furthermore, both pro‐ and anti‐inflammatory cytokines exhibited a tendency to be increased in diseased lesion compared with the healthy controls (Figure 6D). These findings indicated that increased expression of Fc‐gamma receptor‐related genes, resulting from genomic changes contributes to increased inflammation in the affected site.

### 2.5 Validation of the results in single cell RNA sequencing data

**FIGURE 7:**
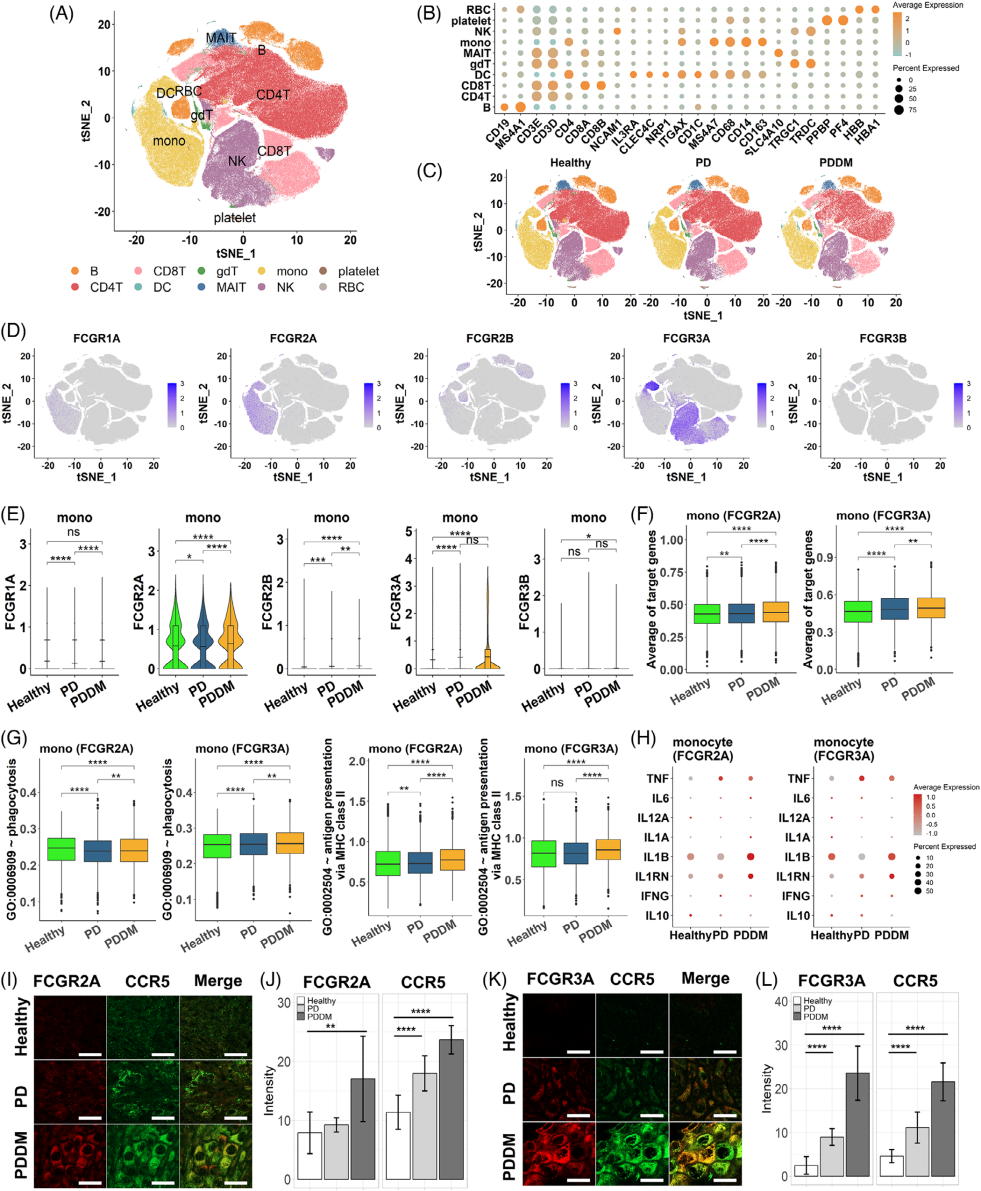
Expression of Fc‐gamma receptor and functional alterations in monocytes at single cell resolution with immunohistological staining in periodontal tissue. (A) The tSNE plot of the integrated dataset. A total of 243,038 cells were included. Each color represents an identity of PBMC cell type. (B) The dot plot describing expression of canonical cell type markers. The size of dot encodes the proportion gene‐expressing cells within a cell type, whereas the color indicates the average expression of genes. The higher and lower level of expression is represented in orange and green colors, respectively. (C) The tSNE plot divided by disease condition. (D) Expression level of Fc‐gamma receptor subunits projected on tSNE plot. (E) The violin plot showing expression level of Fc‐gamma receptors in monocytes. The horizontal line represents average expression for each group and star notation demonstrates significance of difference based on Wilcoxon rank sum test. *p* ≤ 0.05 (*), *p* ≤ 0.01 (**), *p* ≤ 0.001 (***), *p* ≤ 0.0001 (****), *p* ≥ 0.05 (ns). (F) Box plots displaying expression of WGS‐identified hypomethylated genes in monocytes. (G) The expression level of gene set involved in phagocytosis (GO:0006909) and antigen processing and presentation of peptide or polysaccharide antigen via MHC class II (GO:0002504). The activities are calculated in *FCGR2A* or *FCGR3A* expressing monocytes. The horizontal line represents average expression for each status and star notation demonstrates significance of difference based on Wilcoxon rank sum test. *p* ≤ 0.05 (*), *p* ≤ 0.01 (**), *p* ≤ 0.001 (***), *p* ≤ 0.0001 (****), *p* ≥ 0.05 (ns). (H) The cytokine levels of monocytes. The cytokines released by Fc‐gamma expressing monocytes are presented in each dot plot. The higher level of expression is in red while dot size reflects the proportion of cytokine‐expressing cell within a group. (I) Immunohistological staining against FCGR2A and CCR5 in healthy, PD, and PDDM group. Scale bar indicates 25 µm. (J) The immunohistochemistry staining intensity using Image J from healthy (*n* = 3), PD (*n* = 4), and PDDM (*n* = 5) tissue. *y*‐Axis describes the percentage of positive staining intensity of FCGR2A and CCR5. Four distinct regions were measured within each image and Wilcoxon rank sum test were performed to compare intensities of PD and PDDM compared with healthy donors; *p* ≤ 0.05 (*), *p* ≤ 0.01 (**), *p* ≤ 0.001 (***), *p* ≤ 0.0001 (****), *p* ≥ 0.05 (ns). (K and L) Similar to Figure 7I,J, but for FCGR3A and CCR5.

Upon integrating the previous results, we not only observed an increase in phagocytosis mediated by Fc‐gamma receptors in periodontal tissues but also indirectly suggested potential activity in pancreatic tissues. This led us to suspect systemic stimulation of phagocytic activity, subsequently prompting questions about which immune cells make contributions. Hence, we examined changes in the Fc‐gamma receptor‐mediated phagocytosis in the immune cells at single‐cell resolution. We analyzed 243,038 peripheral blood mononuclear cells (PBMCs) from healthy individuals, PD, and PDDM patients. The cell types of PBMCs such as B, CD4T, CD8T, gamma delta T (gdT), dendritic cell (DC), monocyte, and natural killer (NK) cell were identified by the representative cell markers (Figure 7A,B). No batch effect was observed depending on the patient group, as all types of cells were evenly distributed (Figure 7C). The Fc‐gamma receptors were expressed on various immune cells, especially monocytes, DCs, and NK cells in PBMCs (Figure 7D). Since Fc‐gamma receptors can be classified based on their structure and function, we explored the expression level for three subtypes of Fc‐gamma receptors: type I Fc‐gamma receptor (*FCGR1A*), type II Fc‐gamma receptor (*FCGR2A*, *FCGR2B*), and type III Fc‐gamma receptor (*FCGR3A*, *FCGR3B*). The expression analysis revealed that monocytes predominantly expressed *FCGR2A* and *FCGR3A*, while the expression of other receptors were relatively low (Figure 7E). *FCGR3A* was predominantly expressed in NK cells across all three conditions and in DCs, the expression of *FCGR1A*, *FCGR2A*, and *FCGR3A* were significantly increased in patients with PDDM compared with that in the healthy individuals and those with PD (Figure S2A,B). This is consistent with previous studies reporting that the expression of these receptors can be induced under inflammatory stimulation.^32^, ^33^ Intriguingly, in both the PD and PDDM groups, FCGR2A+ and FCGR3A+ monocytes specifically expressed higher level of the targeted genes, than that of the healthy controls. Moreover, the gene expression levels in the PDDM group were higher than that of the PD group (Figure 7F). To investigate the role of intracellular signaling cascades in Fc‐gamma receptor‐expressing cells, we quantified the activity for antigen processing and presentation of peptide or polysaccharide antigen via MHC class II as well as phagocytosis. In FCGR2A/FCGR3A‐expressing monocytes, the antigen‐presenting process was more activated in both disease groups compared with that of the healthy controls, with the highest activation observed in patients with PDDM (Figure 7G). Consistent with this observation, Fc‐gamma receptor‐expressing monocytes expressed higher amounts of pro‐inflammatory cytokines, such as *TNF*, *IL1RN*, and *IFNG*, in PD and PDDM patients compared with those in the healthy individuals. Conversely, IL10 and anti‐inflammatory cytokine expressions were reduced in patients with PD and PDDM (Figure 7H). In other immune cells, such as NK cells and DCs, phagocytosis and antigen presentation were upregulated in the PD group and compromised in the PDDM group (Figure S2C,D). Most of the cytokines exhibited substantial expression in patients with PD, whereas inconsistent expression was observed in patients with PDDM (Figure S2E,F). To verify the key genes at protein level, we conducted immunohistochemistry staining on periodontal tissue, obtained from healthy, PD and PDDM group. The analysis revealed a progressive augmentation in FCGR2A intensities within monocytes expressing CCR5, in contrast to healthy donors (Figure 7I,J). Consistent patterns were also observed for FCGR3A (Figure 7K,L). These consistencies indicate that monocytes play a crucial role in increasing systemic inflammation through the activation of Fc‐gamma receptors. This finding highlights the importance of targeting the signaling pathway in monocytes to reduce the systemic inflammation in both PD and PDDM, especially in terms of diabetic complications.

## 3 DISCUSSION

In the present study, we compared the genetic and epigenetic disparities between patients with PD and PDDM. These findings were validated by comprehensively analyzing transcriptome data. This is the first study to characterize the genomic landscape of these two chronic diseases through the application of multiomics profiling.

Understanding the interplay between genetic and epigenetic factors related to diabetic complications is fundamental to unraveling the disease's pathological mechanisms. Recent studies have highlighted that changes in methylation, an epigenetic mechanism, often intersects with candidate genes identified in genome‐wide association studies, suggesting that genetic variations could increase the risk of T2DM by influencing DNA methylation, thereby impacting pancreatic function.^34^ Notably, these observations were made in pancreatic tissue and blood. In our study, we observed that PDDM was associated with more hypomethylated CpG sites and C>T base substitutions than PD, reinforcing a significant link between these two events. Consequently, we proposed that the synergic effect of both genetic and epigenetic changes may predispose to or worsen diabetic complications, even in susceptible tissues. In addition, there is a higher burden of genetic mutations associated with PI3K–AKT, extracellular matrix (ECM), and T2DM signaling pathway in PDDM. These signaling pathways have been consistently reported to be associated with T2DM in previous studies.^35^, ^36^, ^37^ For instance, the mutations in genes such as *PIK3CA* (phosphatidylinositol‐4,5‐bisphosphate 3‐kinase catalytic subunit alpha) and *AKT1* (AKT serine/threonine kinase 1) can lead to dysregulation of the pathway or alteration of signaling capacity, resulting in the development of insulin resistance and its association with T2DM.^37^, ^38^ In addition, it is noteworthy that PI3K–AKT pathway along with the Fc‐gamma receptors‐mediated pathway identified in hypomethylated gene sets. Both pathways play significant roles not only in cell survival, growth, and differentiation but also in the activation of macrophages.^39^, ^40^, ^41^ Moreover, the PI3K/Akt pathway is recognized to be triggered by Fc receptors, modulating cytokine production.^42^, ^43^ Consistent with these findings, monocytes exhibited elevated phagocytosis through Fc‐gamma receptors in the PD and PDDM as evidenced by single‐cell analysis. The composition of ECM, regulated by various genes, can also impact the onset and progression of T2DM. For example, genetic mutations in the ECM component protein *COL4A3*, *MMP‐9*, and TIMP‐1 have been reported to be associated with T2DM.^44^ It indirectly supports the hypothesis that the development and exacerbation of diabetic complications may result from the invasion of tissues by cells with specific functional impairments. Hence, our results offer new insights and opportunities for further exploration of the mechanisms underlying diabetic complications.

We also identified changes at the individual gene level through the presence of more hypomethylated CpG sites and C>T nucleotide substitutions in PDDM than PD in genome‐wide regions. Interestingly, the genes exhibiting these features were associated with the Fc‐gamma receptor‐mediated phagocytosis signaling pathway. Fc‐gamma receptors, primarily found on immune cells, play a dual role in transmitting both inflammatory and noninflammatory signals.^45^, ^46^ Due to their involvement in various inflammatory diseases, they have been extensively suggested as potential therapeutic targets in immune‐mediated diseases.^47^, ^48^ Though the role of Fc‐gamma receptors in PD is not well understood, polymorphisms in Fc‐gamma‐receptor genes, specifically FCGR2A, FCGR3A, and FCGR3B, are associated with pathogenesis of chronic PD.^49^, ^50^, ^51^ In addition, Fc‐gamma receptor has been implicated as a shared characteristic with PD complications.^52^ On the other hand, the role of Fc‐gamma receptors in T2DM is controversial. Phagocytosis via Fc‐gamma receptors has been reported to be impaired in patients with T2DM.^53^ However, some article have suggested their deficiency attenuates diabetic nephropathy and their activation contribute to the development of autoimmune diabetes.^54^, ^55^ Additionally, our previous analysis of PD and T2DM differential expression revealed a significant upregulation of genes related to the Fc‐gamma receptor‐mediated phagocytosis pathway.^56^ Therefore, our results emphasize once again the importance of the Fc‐gamma receptor‐mediated phagocytosis signaling pathway in T2DM and accumulated C>T mutation and hypo methylation. Although future experiments involving Fc‐gamma receptor inhibition, such as through miRNAs or antibodies, would be needed to determine whether alleviation of PD and DM complications development can be achieved via the Fc‐gamma receptor pathway,^57^, ^58^ these results underscore the importance of genes related to Fc‐gamma receptor‐mediated phagocytosis as potential therapeutic targets. We performed additional validation in this study through expression profiling datasets conducted in periodontal and pancreatic tissues and confirmed upregulated expression values. We further performed single‐cell analysis of PBMCs to identify circulating cells invading the periodontium. As a result, we identified genes related to Fc‐gamma receptor‐mediated phagocytosis that were significantly upregulated in monocytes of PDDM compared with PD and observed prominently elevated protein of the Fc‐gamma receptor in periodontal tissue as well.

Nonetheless, our study has several limitations. While we utilized various omics datasets to validate Fc‐gamma receptor‐mediated phagocytosis‐related genes, including publicly available data from repositories like Gene Expression Omnibus (GEO), inherent differences among datasets may arise due to variations in experimental design, sample populations, and technical platforms. Therefore, while analysis of GEO data provide valuable insights, cautious interpretation needs to be warranted due to limitations in observational data and the potential presence of uncontrolled variables. Additionally, we conducted correlation analysis hypothesizing that cumulative C>T base substitutions would lead to an increase in hypo dmCpG. As anticipated, we observed significant linearity between C>T base substitutions and hypo dmCpG in all PD and PDDM groups. However, as these results are based on correlation analysis, further experimental validation is essential.

## 4 CONCLUSION

In our study, periodontal tissue had significantly more somatic mutations in PDDM. These results and single‐cell analysis indicate the possibility that T2DM‐induced monocytes with C>T base substitutions and hypo‐methylation events invaded the periodontal tissue. Therefore, we believe that targeting the monocyte Fc‐gamma receptor‐mediated phagocytosis signaling pathway as a potential therapeutic target could be a promising treatment method to inhibit T2DM and its related complications.

## 5 MATERIALS AND METHODS

### 5.1 Human sample acquisition and criteria

This research was carried out following the principles outlined in the Declaration of Helsinki, with approval obtained from the Institutional Review Board of Pusan National University Dental Hospital (IRB No. PNUDH‐2020‐032). Verbal and written consent was obtained from all the study patients prior to the commencement of the study regarding the use of their blood and tissue samples. Individuals were selected and classified into healthy group, PD group, and PDDM group on the basis of their medical history, which was obtained through interview and questionnaire. Patients with <3 mm of pocket depth and no clinical manifestation of inflammation, including swelling, redness, or bleeding upon probing, were regarded as healthy individuals. Hb1AC levels were measured and periodontal status was assessed to verify the presence of PD and PDDM. Individuals with systemic illnesses apart from DM and those who received periodontal treatment or antibiotics in the past 6 months were not included in the study. Venous blood was collected through regular venipuncture and stored in plastic tubes containing ethylenediaminetetraacetic acid. A gingival tissue, comprising the pocket epithelium and connective tissue, was collected from each patient. These tissues were gathered during periodontal flap surgery from individuals diagnosed with PD and PDDM. For subjects who are included in healthy group, tissue collection took place during procedures such as crown lengthening or extractions unrelated to periodontal issues. Follwing extraction, the gingival tissue underwent washing with sterile normal saline solution to eliminate any blood clots or detached plaque adhering to the tissue surface.

### 5.2 PBMC Isolation

PBMCs were separated within 30 min of collection using SepMate (Stemcell Technologies, Seattle, WA, USA) according to the manufacturer's instructions. The procedure involved adding the insert with density gradient medium, filling the SepMate tube with diluted blood sample mixed with phosphate‐buffered saline (PBS) and 2% fetal bovine serum (FBS) and then centrifuging at room temperature at 1200×*g* for 10 min. The upper layer was transferred to new tubes, and subjected to two washes with PBS and 2% FBS, followed by another centrifugation at 120×*g* for 10 min at room temperature. The obtained PBMCs were then frozen and stored at −80°C before use.

### 5.3 MGI PE library construction and sequencing

The genomic DNA underwent fragmentation, resulting in DNA fragments ranging from 100 to 1000 base pairs, ideal for PE150 sequencing. This fragmentation process was carried out using the Frag enzyme from MGI in Shenzhen, China, following the guidelines provided by the manufacturer's MGI FS DNA library prep set (cat No. 1,000,005,256). The fragmented DNA was further selected using DNA clean beads (MGI, Shenzhen, China) so that it ranged between 300 and 500 bp. Following fragment selection, the DNA fragments underwent repair to achieve a blunt end and were altered at the 3′ end to produce a dATP sticky end. Subsequently, both ends of the DNA fragments were attached with a dTTP‐tailed adapter sequence through ligation. The resultant ligation product underwent seven cycles of amplification and was then subjected to a single‐strand circularization procedure. This involved heat‐denaturing the polymerase chain reaction (PCR) product alongside a complementary molecule reverse‐complemented to a specific strand of the PCR product, followed by ligating the single‐strand molecule using DNA ligase. The residual linear molecule was digested with exonuclease, resulting in the formation of a single‐strand circular DNA library. The sequencing of this DNA library was carried out using DNBSEQ‐T7 with a paired‐end read length of 150 base pairs.

### 5.4 Whole‐genome sequencing analysis

The whole‐genome sequences were analyzed based on the Genome Analysis Toolkit (GATK) Best Practices for short somatic variant discovery.^59^ The procedure included mapping sequence reads to the human reference genome (GRCh38) using the Burrows‐Wheeler Aligner MEM algorithm, identification and handling of duplicates using Picard, and recalibration of base quality scores using GATK.^60^ We achieved a 15‐fold and 50‐fold coverage for 79−99 and 42−92% of the target bases, respectively, with a minimum base quality score of 20. We applied Mutect2 in GATK4 to identify somatic variants, using a “panel of normals” created from 1000 Genomes patients and the gnomAD database as a “germline‐resource.” We included in our analysis all somatic variant calls that met the standard Mutect2 filters criteria. We ran Mutect2 in “tumor‐only” mode, disregarding the “germline_risk” filter. Any variant calls that solely relied on the “germline_risk” filter were not included in our analysis. The resulting somatic variant calls were then annotated using FUNCOTATOR of GATK4.

### 5.5 EPIC array bioinformatics analysis

The genomic DNA underwent bisulfite conversion utilizing the EZ DNA methylation kit from Zymo Research. Subsequently, it was examined using the Illumina Infinium MethylationEPIC BeadChip, following the manufacturer's instructions. The analysis was conducted on the iScan system by Illumina at Queensland University of Technology. The raw data, comprising IDAT files from 37 samples, underwent importation, filtering, and normalization utilizing the ChAMP package within R software (version 3.5.1).^61^ Initially, probes were excluded if their detection *p* value was ≥0.01, or if they showed fewer than three beads in 5% or more of the samples. To address biases inherent in probe types 1 and 2, β‐mixture quantile normalization was conducted, followed by standard quantile normalization. As a result of this filtering process, 128,687 probes were eliminated, leaving 684,246 probes for subsequent analyses. A singular value decomposition analysis revealed the absence of substantial batch effects across methylation arrays and slides. To pinpoint individual CpG sites displaying distinct DNA methylation patterns specific to PD and PDDM, we conducted a comparative methylation analysis between the two conditions. This involved employing *t*‐tests for 684,246 probes, with adjustments made for multiple testing using the Benjamini–Hochberg method within the R function p.adjust. Significance was determined if the adjusted *p* value was ≤0.05, and if the disparity in average beta values between the two groups was at least 0.2.

### 5.6 Library construction and processing of single cell RNA‐seq

scRNA‐seq libraries were generated using the chromium controller, following the 10× chromium Next GEM Single Cell 3′ v3.1 protocol. The cell suspension was combined and applied onto Single Cell 3′ v 3.1 Gel Beads to generate the cDNA library. Subsequently, the library was amplified and subjected to sequencing using the Illumina HiSeq platform. The scRNA‐seq data were generated using 10× Genomics Cell Ranger v5.0.1, and FASTQ files were processed using the “cellranger mkfastq” command.^62^ The raw FASTQ files were aligned in accordance with the human reference genome (GRCh38, v3.0.0) and a gene expression matrix was generated with a unique molecular identifier (UMI) and cell barcode, using “cellranger count” command. The produced count data were loaded, combined, and preprocessed using Seurat v4.1.1 22. To avoid doublet, we used DoubletFinder for each sample and removed the estimated cells 23. Further, cells with less than 500 UMIs or more than 15,000 UMIs and more than 15% mitochondrial reads were discarded. Cells were retained if they had at least 250 genes and no more than 5000 genes. The filtered dataset was normalized using sctransform and integrated by specifying one reference data in healthy individuals, on the basis of number of cells. To visualize the integrated data, dimension reduction was conducted using principal component analysis and t‐distributed Stochastic Neighbor Embedding (tSNE). The first 20 principal components were used to construct shared nearest neighbor modularity optimization and Louvain algorithm for community detection was applied to identify cell clusters.

### 5.7 Identification of PBMCs

Canonical markers of PBMCs were inspected to reveal the identity of clusters. These included CD19, MS4A1 (B cell), CD3E, CD3D (T cell), CD4 (CD4T cell), CD8A, CD8B (CD8T cell), NCAM1 (NK cell), IL3RA, CLEC4C, NRP1, ITGAX, CD1C (DC), MS4A7, CD68, CD14, CD163 (monocyte), SLC4A10 (Mucosal‐associated invariant T cell; MAIT), TRGC1, TRDC (gamma‐delta T cell; gdT), PPBP, PF4 (platelet), and HBB, HBA1 (red blood cell).^63^, ^64^

### 5.8 Gene list from Gene Ontology and statistical analysis

To explore the functionality of phagocytosis and antigen presentation in each cell type, the average of gene expression for corresponding gene set was calculated per cell. Gene lists for phagocytosis (GO:0006909) and antigen processing and presentation of peptide or polysaccharide antigen via MHC class II (GO:0002504) were obtained from the Molecular Signatures Database (MSigDB).^65^ The significance of difference between two groups was determined using nonparametric two‐sided Wilcoxon rank sum test. *p* Value < 0.05 was considered statistically significant.

### 5.9 Immunohistological analysis

For immunohistochemistry, paraffine‐embedded tissue sections were deparaffinized, followed by incubation with 3% hydrogen peroxide for 15 min to block the endogenous peroxidases. Next, the sections were blocked with 1% bovine serum albumin for 30 min and then incubated with antibodies against FCGR2A (Abcam), FCGR3A (Abcam), and CCR5 (Santacruz) for overnight at 4°C. The sections were then stained with goat anti‐rabbit IgG H&L (Alexa Fluor® 594; Abcam) and goat anti‐mouse IgG H&L (Alexa Fluor® 488; Abcam) for 3 h. Confocal images were analyzed using a Carl Zeiss LSM 800 laser scanning microscope, the Zen Blue program (magnification: ×200) and Image J software.

### 5.10 Statistics analysis

The statistical analyses were performed under R Environment v4.1.2. The Wilcoxon rank‐sum test (for two groups) or Kruskal–Wallis test (for more than two groups) was examined to explore differences between groups. We utilized the Fisher's exact test to evaluate the overrepresentation of mutations within a specific gene compared with the baseline mutation frequency. All analyses were conducted with two‐sided tests, and statistical significance was determined at *p* values ≤0.05. Multiple testing adjustments was done using the Benjamini–Hochberg False Discovery Rate method and was employed to differential gene‐methylation and GO enrichment analyses.

## AUTHOR CONTRIBUTIONS

Yun Hak Kim, and Hae Ryoun Park designed and supervised the study. Junho Kang, Hansong Lee, and Yun Hak Kim performed computational analysis and drafted and revised the manuscript. Ji‐Young Joo, Jae‐Min Song, Hyun‐Joo Kim, and Hae Ryoun Park collected patient consent for the study and conducted experiments. Junho Kang, Hansong Lee, Ji‐Young Joo, Jae‐Min Song, Hyun‐Joo Kim, Yun Hak Kim, and Hae Ryoun Park discussed the results. All authors have read and approved the final manuscript.

## CONFLICT OF INTEREST STATEMENT

The authors declare no conflict of interest.

## ETHICS STATEMENT

This study was conducted in accordance with the tenets of Declaration of Helsinki after obtaining approval from the Institutional Review Board of Pusan National University Dental Hospital (IRB No. PNUDH‐2020‐032). Informed consent was obtained from all patients for being included in the study.

## Supporting information

Supporting information

Supporting information

## Data Availability

The WGS, EPIC array, and scRNA‐seq datasets supporting the conclusions of this study are available from the SRA and GEO repositories with accession numbers PRJNA1116808, GSE267170, and GSE244515, respectively.
