# Supplementary material for: Comparison of genetic and epigenetic profiles of periodontitis according to the presence of type 2 diabetes
Source: MedComm (2020). 2024 Jun 19;5(7):e620. doi: 10.1002/mco2.620 (PMC11187843; doi:10.1002/mco2.620)
Supplement: Supplementary file 1 — Supporting information [file MCO2-5-e620-s002.docx]

Title: Comparison of Genetic and Epigenetic Profiles of Periodontitis According to the Presence of Type 2 Diabetes

**List of Supplementary Materials**

**Table S1. List of SNPs identified in PD.**

**Table S2. List of SNPs identified in PDDM.**

**Table S3. The results of functional enrichment analysis through high mutational burden genes.**

**Table S4. The results of functional enrichment analysis through hypo dmCpG and C>T base substituted genes.**

**Fig. S1. Gene plot of genes associated with C>T base substitution and hypo dmCpG.**

**
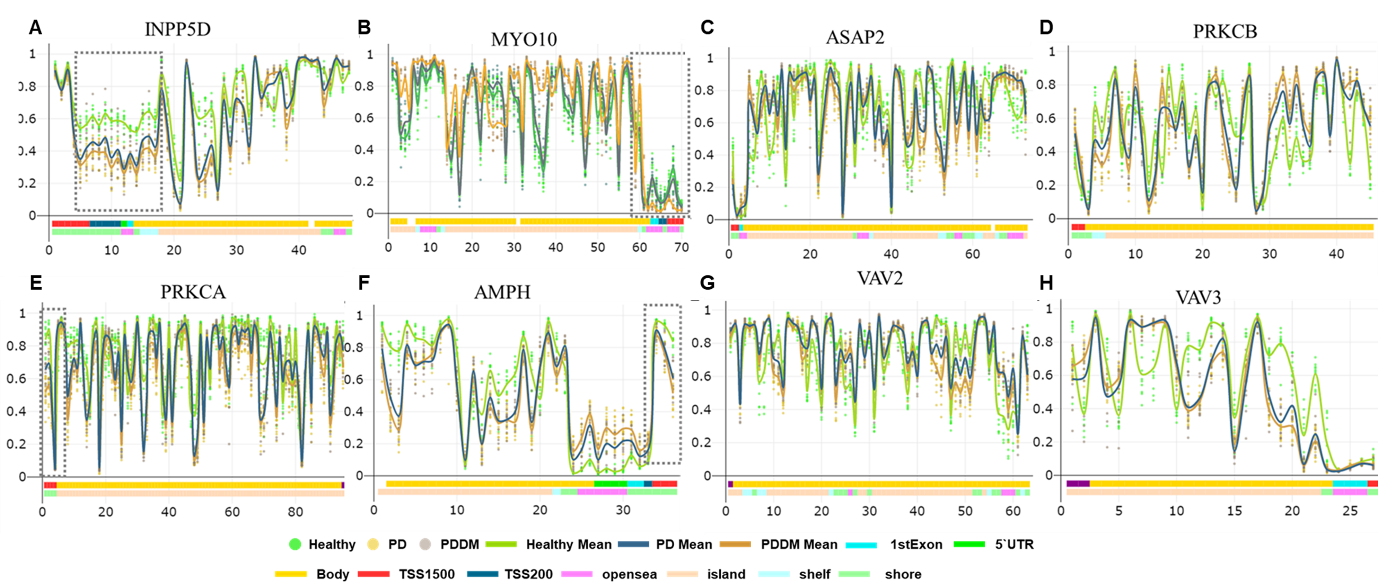
**

Each line and color correspond to the legend displayed on the right side. The x-axis represents the absolute position in the gene, and the y-axis represents the methylation intensity. Dotted lines indicate regions where C>T base substitutions and hypomethylation coincide. A: *INPP5D*. B: *MYO10*, C: *ASAP2*, D: *PRKCB*, E: *PRKCA*, F: *AMPH*, G: *VAV2*, H: *VAV3.*

**
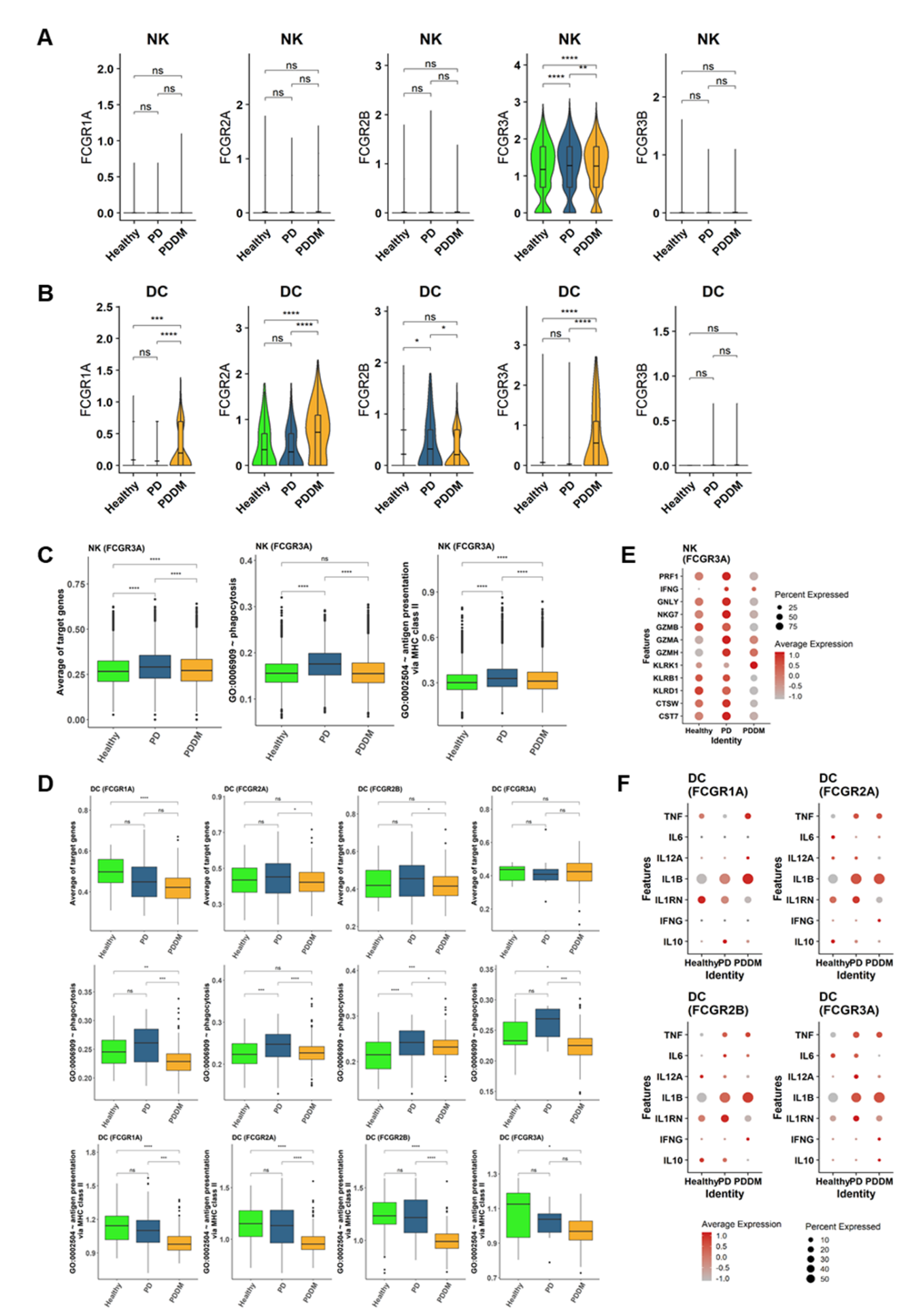
Fig. S2. The activity of phagocytosis and antigen presenting in NK cells and DCs.**

**A-B.** The violin plot showing expression level of Fc-gamma receptors in NK cells **(A)** and DCs **(B)**. The horizontal line represents average expression for each group and star notation demonstrates significance of difference based on Wilcoxon rank sum test. *p* ≤ 0.05 (*), *p* ≤ 0.01 (**), *p* ≤ 0.001 (***), *p* ≤ 0.0001 (****), *p* ≥ 0.05 (ns). **C-D.** The expression level of gene set involved in phagocytosis (GO:0006909) and antigen processing and presentation of peptide or polysaccharide antigen via MHC class II (GO:0002504) in NK cells **(C)** and DCs **(D)** expressing Fc-gamma receptor subunit. The horizontal line represents average expression for each status and star notation demonstrates significance of difference based on Wilcoxon rank sum test. *p* ≤ 0.05 (*), *p* ≤ 0.01 (**), *p* ≤ 0.001 (***), *p* ≤ 0.0001 (****), *p* ≥ 0.05 (ns). **E-F.** The cytokine levels of NK cells **(E)** and DCs **(F)**. The corresponding cytokines released by NK cells and DCs are presented in each dot plot. The higher level of expression is in red while dot size reflects the proportion of cytokine-expressing cell within a group.
